# Rethinking the Prevention of Cognitive Decline in Healthy Older Adults: The Motor-Cognitive Active Gamified Immersive Cultural (MAGIC) Exergames

**DOI:** 10.2196/100013

**Published:** 2026-09-30

**Authors:** Jean-Jacques Temprado

**Affiliations:** 1 Institute of Movement Science, Aix-Marseille Université, 163 Avenue de Luminy - BP 910, Marseille, Provence-Alpes-Côte d'Azur, 13288, France

**Keywords:** aging, exergames, cognition, neuroaesthetics, exercise snacking, immersive cultural experience

## Abstract

Though exergames attract considerable research interest as tools for preserving cognitive function in older adults, a decade of meta-analytic evidence reveals a persistent gap between theoretical promise and empirical demonstration: exergames rarely outperform conventional motor-cognitive training and do not generate the sustained engagement that preventive benefit requires. These results reflect limitations that are structural: the field focused predominantly on clinical populations rather than primary prevention; training concepts borrowed from rehabilitation protocols rather than grounded in the specific neuroplastic mechanisms of the exergaming medium; and virtual environments that are digitally sophisticated but culturally and esthetically neutral, systematically excluding the stimulation that converging evidence from neuroaesthetics, cultural epidemiology, and narrative neuroscience identifies as a core active ingredient of brain health promotion. To address these limitations simultaneously, this viewpoint introduces Motor-Cognitive Active Gamified Immersive Cultural (MAGIC) exergames, defined as “virtual reality solutions in which motor-cognitive interactions are embedded in artistic content, cultural heritage, or historically situated narrative to exploit optimal neurobiological substrates of the intervention.” Drawing on an integrated theoretical framework, we develop mutually reinforcing mechanistic pillars: multimodal sensorimotor engagement within ecologically valid immersive environments; semantic and narrative activation of memory systems; emotional and autobiographical resonance as a dopaminergic driver of learning consolidation and sustained participation; and the re-engineering of physical effort evaluation from aversive to appetitive through aesthetic pleasure and narrative absorption. A fundamental implication is that in MAGIC exergames, motivation is not engineered through gamification mechanics—it is released by the intrinsic value of inhabiting a culturally meaningful world. We further argue MAGIC exergames correspond to a specific use that is, Cultural Snacking, which consists of the serialization of MAGIC content into brief, narratively open daily episodes of 5 to 10 minutes, designed for 3 to 5 daily consumptions across varied contexts. This dosing architecture is compatible with the motivational profile of healthy older adults who do not identify as patients or trainees. The paper opens perspectives with a design and research agenda structured around three priorities: characterizing MAGIC exergames by the type of motor-cognitive interactions they deliver rather than by the technology platform; conducting mechanistic neuroimaging investigations targeting the specific biomarkers of neuroplastic benefit that creative cultural engagement is expected to produce; and building the intersectoral innovation ecosystem that brings cultural institutions, digital health teams, and older adults together as co-designers. Without waiting for MAGIC-specific trials, we invite the digital medicine, public health, and silver economy communities to recognize that museums, heritage sites, and cultural institutions are potentially the most ecologically valid, intrinsically motivating, and cost-effective substrates for a new generation of brain health exergames that are as meaningful as they are effective. The scientific, institutional, and economic conditions for this convergence already exist.

## Exergames, Brain Health, and Cognition: A Promising Yet Unfulfilled Agenda

As populations age at unprecedented rates, primary prevention of cognitive decline and neurocognitive disorders has become a strategic priority of public health systems worldwide, with the aim of preserving the brain health of healthy older adults before clinical thresholds are reached [1]. Older adults are acutely aware of the prospect of cognitive decline, but evidence-based options available to them remain largely confined to aerobic and muscular exercise programs [2], that many people find insufficiently motivating to sustain. This adherence problem is structural. Devices developed within a clinical or rehabilitation context benefit from externally supported adherence: medical prescription, professional supervision, a therapeutic framework, and an explicit recovery goal collectively sustain engagement, so that motivation does not need to be intrinsically generated by the intervention itself. This support structure is absent in primary prevention. Among healthy individuals who present no symptoms, face no institutional obligation, and answer to no supervisor, adherence can rest on nothing but intrinsic interest in the activity. This contextual shift is fundamental, and it constitutes, we argue, the central justification for the Motor-Cognitive Active Gamified Immersive Cultural (MAGIC) framework developed in this paper: designing a desirable, culturally meaningful environment that fits naturally into daily life is not a cosmetic optimization of an exergame intervention—it is the condition of its preventive effectiveness.

Over the past decade, exergames—that is, virtual reality solutions that combine physical activity with cognitive challenge within a gamified interface—have been considered as a promising solution for preventing, delaying, or compensating (at least partially) brain alterations and decrease in cognitive performance. By simultaneously delivering cardiovascular effort (endurance), complex goal-oriented movements, and cognitive stimulation in gamified virtual environments, they should, in theory, be more motivating and produce synergistic neuroplastic effects superior to single-domain interventions. Meta-analyses and systematic reviews have reported benefits in executive function, memory, and processing speed [3-5], yet a more rigorous reading of the cumulative evidence reveals a persistent gap between theoretical promise and empirical demonstration [6-9]. For instance, the first and most influential meta-analysis in the field, Stanmore et al [4], reported a moderate positive effect of exergames on global cognition, yet no evidence was found that exergames outperform cognitively demanding control conditions. Subsequent analyses have extended and deepened this assessment. Sokolov et al [7] found no significant advantage of exergames over conventional exercise for cognitive outcomes when head-to-head comparisons were made under controlled conditions, challenging the central claim that the combination of physical and cognitive stimulation in a gamified interface produces synergistic neuroplastic effects. Generally, the meta-analytic literature on exergames and cognition displays a pattern that is characteristic of confirmation bias: effect sizes consistently diminish as methodological rigor increases, and the findings most frequently cited as proof of concept contain, upon closer reading, the seeds of more uncertain conclusions. This conclusion is compounded by a well-documented publication bias, that is, selective reporting of positive findings; once publication bias is corrected for, the apparent cognitive benefits are substantially attenuated [6].

However, despite these conclusions, the field is maintaining a firm optimism about the cognitive benefits of exergaming [10,11], which is a direct consequence of the belief in the magical power of technology and the “product-first” approach, testing specific commercial platforms designed for entertainment as if the product itself were the intervention, rather than characterizing the type of combined training delivered. In a systematic review specifically designed to characterize the type of combined training delivered by exergames, Torre and Temprado [9] identified that the overwhelming majority of exergame studies do not characterize and control for the actual training content delivered, regardless of the technological interface. By misidentifying the exergame as the unit of analysis rather than the training program it supported, the field has systematically generated a literature too heterogeneous to be informative and too methodologically flawed to support causal inference. This failure has limited the understanding of the mechanisms engaged to enhance brain plasticity and cognitive performance in durable and transferable ways [12,13], so that whether and how exergames can be meaningfully improved remains an open question.

## How Can Exergames Better Enhance Brain Health and Cognition in Older Adults?

The design logic of exergames required to better enhance brain health and cognition should overcome the limitations that have constrained exergame research in the discovery of effective neuroplastic principles. Four design recommendations follow directly from this diagnosis. First, new exergames should not limit themselves to a curative objective. Instead, the entire design logic—environments, task demands, and motivational architectures—should be structured for the primary prevention of cognitive decline in cognitively healthy older adults, that is, to preserve and enrich capacities rather than to remediate clinical deficits. This reorientation implies a fundamentally different user model, in which the older adult is not a patient to be treated but a capable individual whose neuroplastic resources are to be maintained and deepened through intrinsically motivating engagement. Throughout this paper, “cognitively healthy older adults” refers operationally to community-dwelling individuals, typically aged 65 to 85 years, without a diagnosed neurocognitive disorder and retaining full autonomy in activities of daily living; this population is distinct from, and considerably larger than, the clinical or prefrail populations that conventional exergame research has predominantly targeted. Specifying outcomes of interest for this population is less straightforward than in rehabilitation, where the therapeutic target and a success criterion can be defined a priori. In primary prevention, the relevant question is not whether performance improves in absolute terms but whether decline relative to an expected age-matched trajectory is slowed or arrested over a multiyear horizon, evaluated against domain-specific cognitive measures and, where available, neuroimaging-derived markers of functional brain aging. We return to this question of outcome specification in the research agenda below. Second, future exergames should be grounded in theoretically specified training concepts that exploit what is genuinely specific to the exergaming medium. Rather than developing motor-cognitive, physical-cognitive, or multidomain training concepts validated against neuroplastic mechanisms, the field has oscillated between 2 equally under-specified approaches: the gamification of existing rehabilitation protocols, principally to increase patient motivation [14], and the adoption of commercial entertainment exergames [15,16]. However, neither approach deeply addresses the questions that a principled exergaming intervention must answer first: what type of movement, what cognitive demand, and what interaction between the two is neurobiologically productive? A related and underappreciated gap concerns the minimum intensity of physical effort, or alternatively the minimum complexity of motor movements, required to exploit facilitation-guidance mechanisms, which remains poorly specified [17,18]. Indeed, the field has largely imported conventional physical activity recommendation logic without questioning whether this dosing architecture is either optimal or realistic for cognitively healthy older adults whose motivational profile and daily routines are poorly served by the scheduled session model. Third, exergame research should capitalize more systematically on the video game literature, which has accumulated robust evidence that regular practice of action video games produces measurable and transferable benefits on attention, processing speed, executive function, and visuo-spatial cognition. This is a body of evidence that health-oriented exergame researchers have systematically ignored, though it provides both a source of theoretical insight into the mechanisms of game-based cognitive enhancement and a design benchmark against which the specific added value of physical and motor engagement in exergames could be rigorously evaluated. Ignoring it has left the field unable to answer a fundamental question: what does motor engagement specifically contribute, over and above the cognitive stimulation that commercial video games already provide? This question is not merely theoretical. Regularly engaging a cognitive process to maintain or strengthen it is also, at least implicitly, the logic underlying cognitive or “brain” training, whose promises and limitations are well-known regarding generalization of training-related benefits beyond the trained task [19,20]. In the present paper, we posit a new framework relative to this corpus: where brain training relies on the repeated, isolated engagement of a single trained process, MAGIC exergames hypothesize that benefit arises from the simultaneous, multisystem engagement of sensorimotor, semantic, and emotion-valuation networks within a single ecologically valid task. Whether this multisystem engagement yields more generalizable benefit than single-process training remains to be established and constitutes one of the central empirical questions this paper’s research agenda is designed to address. Fourth, new exergames should actively exploit the neuroplastic potential of rich cultural, narrative, and autobiographically resonant content rather than designing virtual environments that mimic superficially senior-friendly aesthetics while remaining culturally neutral. The neuro-aesthetic evidence demonstrates that genuine aesthetic and cultural experiences simultaneously recruit sensorimotor, emotion-valuation, and meaning-knowledge neural systems in a configuration that no purposive cognitive task can replicate [21]. The brain clock framework shows that creative engagement delays brain aging in domain-independent ways [22], and the cultural epidemiology literature documents that regular cultural participation predicts slower cognitive decline across decades of longitudinal follow-up [23]. We hypothesize that embedding motor-cognitive training within culturally meaningful, esthetically rich, and narratively coherent environments is more than a cosmetic design choice; it constitutes one of the most promising design decisions available, pending dedicated empirical validation. Since no available exergame considers all these blind spots simultaneously, the principles that make exergaming effective to deliver on its neuroplastic promise remain poorly understood. Thus, the question arises of whether a fundamentally different approach to designing new exergames might succeed where most of the existing ones did not.

Recently, a recommended direction has been to move “beyond just-for-fun exergames” [24], to adopt rigorous step-by-step design processes [25,26], and a logic grounded in scientifically validated training concepts to target the specific mechanisms that underlie, combined training effects [8,13,24]. Though this approach represents a step forward relative to purely entertainment-driven exergames, it often results in libraries of motor-cognitive exercises that mimic therapeutic protocols, simply apply recommendations for conventional physical activity programs, and whose motivational appeal rests almost entirely on the thin scaffolding of gamification. Moreover, the “beyond just for fun approach” converges on the dominance of the facilitation-guidance model of brain-body interaction, either in its neurobiological formulation [27,28] or its more recent motor-cognitive variant that is, the Adaptive Capacity Model (ACM) [29]. Though very heuristic, this model is incomplete since it frames the body as a vehicle for stimulating brain plasticity rather than as the primary site of intelligent engagement with a meaningful world. Also, it considers cognitive training tasks as inputs to be optimized rather than as dimensions of a richer, culturally embedded experience. To address this issue, the present Viewpoint paper introduces a new category of preventive digital intervention and develops the theoretical and empirical foundations for a research and design agenda of these new digital brain-health solutions targeting healthy older adults.

## A New Theoretical Foundation: MAGIC Exergames

The three structural blind spots identified above point toward a category of preventive digital interventions that: (1) target cognitively healthy older adults, (2) are grounded in a principled understanding of neuroplastic mechanisms rather than in the replication of existing exercise protocols, and (3) place culturally rich, esthetically meaningful, and narratively immersive content at the center of the experience. We propose to call this category MAGIC exergames and define it as: “a virtual reality solution in which brain-mind-body interactions are included in artistic content, cultural heritage, or historically situated narrative, to exploit the neurobiological, psychological and emotional mechanisms involved in problem-solving under changing conditions and constraints.” The theoretical orientation underlying MAGIC exergames draws on two converging frameworks. The first is Bardy Human Movement Intelligence (HMI) [30], and the second is the theory of cognizant action developed by Araújo et al [31] within the Ecological Dynamics tradition [32]. Both frameworks share the common premise that movement is not a delivery mechanism for neuroplastic stimulation that ultimately enhances decomposed and isolated cognitive processes, but the medium through which biological, psychological, emotional, and sensorimotor processes are integrated in a high-order dialogue between brain, body, and environment. In this view, cognition does not follow from movement; it is expressed through it. This perspective extends the ACM [29], which accounts for the neurocognitive and physiological bases of the relationships between physical activity, complex motor skills, brain plasticity, and cognition. Where the ACM grounds the benefits of motor-cognitive training in evolutionary neuroscience, HMI and Ecological Dynamics frameworks extend this foundation by incorporating the esthetic, cultural, social, and technological dimensions of the environments in which motor behavior takes place [30]. In doing so, a conceptual continuity is established between multidomain training [9], grounded in the evolutionary neuroscience model, and the richer theoretical architecture required to account for goal-directed movement in informationally dense, culturally and aesthetically meaningful environments. This reframing implies that cognition is inherently embodied, situationally grounded, culturally shaped, and inseparable from the emotional and aesthetic dimensions of the environment in which it unfolds—conditions that generic exergame environments, culturally neutral by design, systematically fail to provide. Applied to the exergame context, this suggests that the design of MAGIC exergames must immerse both the cognitive functions to train and the physical exercises to gamify within the richness and meaning of the environment itself: its affordances, its narrative coherence, its aesthetic resonance, and its capacity to invite the kind of fully embodied, intrinsically motivated engagement from which neuroplastic change most reliably emerges. This is precisely what MAGIC exergames are specifically designed to instantiate: the conservation of intentionality in informationally rich and culturally meaningful environments by coupled perception and action.

## MAGIC Exergames: Design Considerations

The theoretical framework developed above has direct implications for the design of exergames that go beyond the standard recommendations of exercise and medical science to converge toward the capacity of arts, cultural, and creative engagement to prevent ill health, promote well-being, and support cognitive resilience across the lifespan [33,34]. MAGIC exergames instantiate this notion by making the cultural experience a neurobiological and motivational substrate of brain health prevention. Below, we present the different mechanistic pillars of the MAGIC exergames (Figure 1) and different design principles that make them genuinely distinct from both conventional exergames and existing immersive cultural experiences (Table 1). This distinction matters because cultural content could plausibly act through 2 nonequivalent pathways. The first is a neurobiological one: authentic aesthetic experience is hypothesized to jointly recruit sensorimotor, emotion-valuation, and meaning-knowledge systems in a configuration that purposive cognitive tasks and culturally neutral gamified environments do not reproduce. The second is purely motivational: cultural framing functions as a sophisticated wrapper that overrides the automatic cost evaluation described by the Theory of Effort Minimization in Physical Activity (TEMPA) without engaging any neural mechanism beyond those already recruited by the underlying motor-cognitive task. We hold that MAGIC exergames are designed to engage both pathways simultaneously and that their distinctiveness as a category, rather than a culturally themed variant of existing exergaming, rests on this dual and synergistic engagement. Thus, cultural content is not merely dressing applied over a validated cognitive-motor task to make it more palatable, but is hypothesized to codetermine, jointly with the motor-cognitive interaction itself, the neuroplastic outcome. This is a testable claim, and we return to it in the research agenda below.

**Figure 1.**
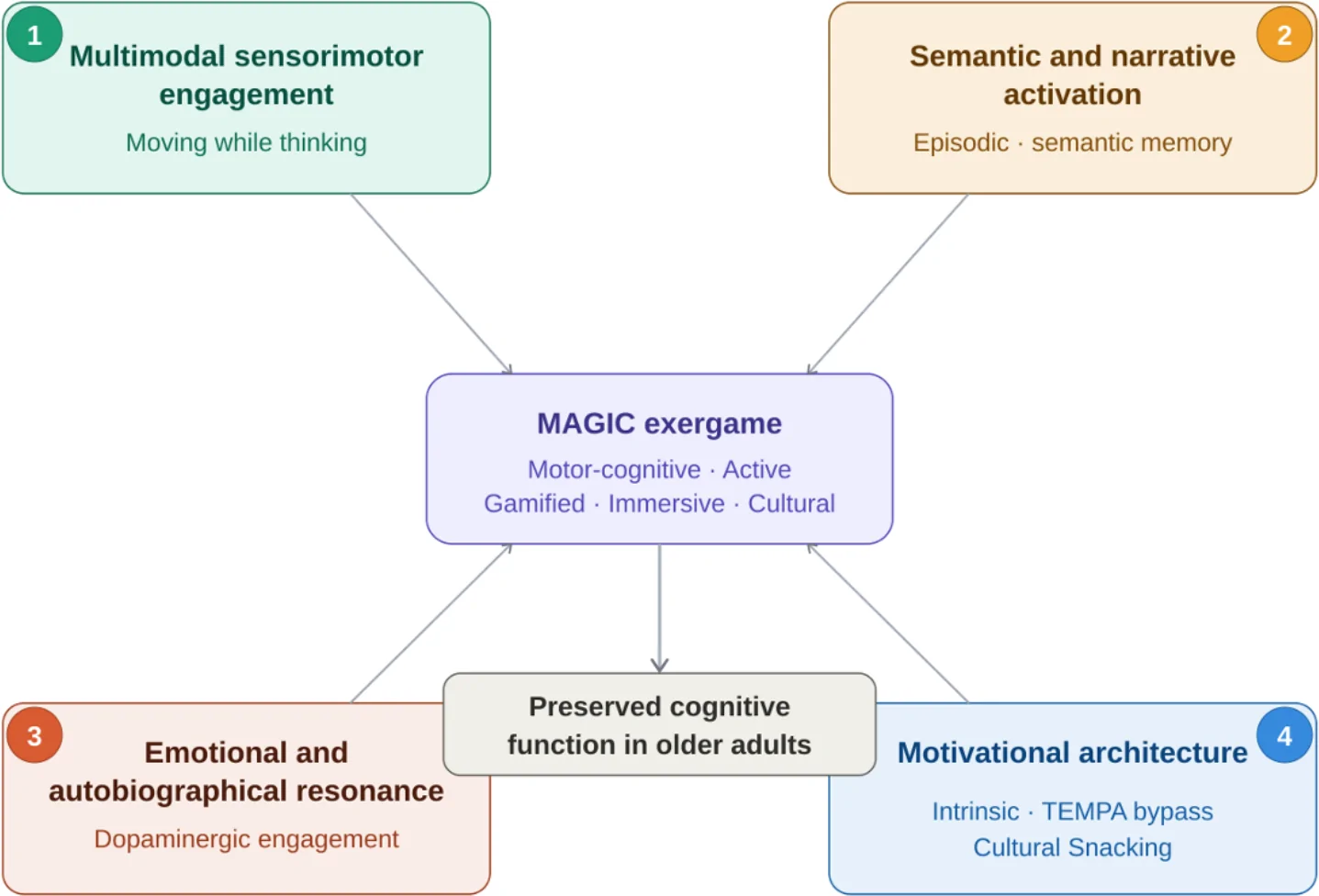
The 4 mechanistic pillars of Motor-Cognitive Active Gamified Immersive Cultural (MAGIC) exergames and their convergence toward the preservation of cognitive function in healthy older adults. Pillar 1 (multimodal sensorimotor engagement) draws on the Adaptive Capacity Model and the Human Movement Intelligence framework to specify the types of motor-cognitive interactions most productive for neuroplasticity. Pillar 2 (semantic and narrative activation) engages episodic and semantic memory systems through culturally coherent narrative environments. Pillar 3 (emotional and autobiographical resonance) exploits dopaminergic reinforcement via personally meaningful cultural content. Pillar 4 (motivational architecture) operates through intrinsic motivation and the Theory of Effort Minimization in Physical Activity (TEMPA) bypass, operationalized as Cultural Snacking—brief, narratively open episodes designed for multiple daily consumptions. Together, these 4 pillars converge to support the preservation of cognitive function in healthy older adults through sustained, intrinsically motivated cultural immersion.

**Table 1.** Examples of Motor-Cognitive Active Gamified Immersive Cultural (MAGIC) interactions across the 3 engagement pathways, specified by the type of motor-cognitive interaction they deliver rather than by platform^a^.

Engagement pathway	Example MAGIC interaction	Underlying motor-cognitive demand (HMI^b^ framework)
Acti-gaming (curiosity-driven)	Wandering a reconstructed 1920s street market to locate a specific vendor’s stall, identified only by a half-remembered visual cue from an earlier episode	Incidental locomotion and visual search under low cognitive load; episodic memory retrieval cued by environmental context
Exergaming proper (sustained return)	Restoring a damaged fresco panel by reproducing, with the hand controller, the gesture sequence of the original painter as revealed progressively across sessions	Bimanual coordination and motor sequence learning, with progressive task complexity invisibly tied to narrative deepening across sessions
Exergame-based training (explicit improvement goal)	Guiding a historical figure safely across a collapsing archive under time pressure, requiring rapid target pointing while withholding response to decoy objects	Speed-accuracy constrained pointing (Fitts-type) combined with response inhibition under time pressure—a moving-while-thinking architecture (stop-signal paradigm)

a Engagement pathways follow the motivational taxonomy proposed by Manser et al [24]. The HMI framework [31] specifies motor-cognitive demands in terms of perception-action coupling within a structured task environment rather than as isolated cognitive processes. All three examples can be delivered on the same platform (eg, a recumbent-bike exergame with a head-mounted display); the pathway distinction is motivational and design-level, not technological.

b HMI: Human Movement Intelligence.

## Multimodal Sensorimotor Engagement and Immersive Presence

In the theoretical framework of MAGIC exergames, neuroplastic change emerges from the continuous, adaptive negotiation between a moving body and an informationally rich environment. High-fidelity immersive environments in three-dimensional virtual reality recruit a broader and more ecologically valid range of sensorimotor processes than two-dimensional screen-based exergames. Therefore, the sense of presence becomes a central mechanism that simultaneously engages proprioceptive, vestibular, and peripersonal space systems, creating the conditions for motor-cognitive interaction at a scale that flat-screen interfaces cannot achieve. Moreover, when cultural content structures the immersive environment, guiding participants through a virtual reconstruction of a heritage site, animating historical artifacts, and embedding movement cues within a narrative, the sensorimotor engagement acquires the additional quality of contextual relevance. Actions have meaning within the story: the player does not feel they move to fulfill a training prescription but to navigate a world through goal-directed behaviors whose significance is immediately legible. Such an engagement of motor-cognitive interactions moves beyond classical dual-task paradigms toward what Herold et al [35] term “Moving while Thinking” situations, in which cognitive tasks are incorporated into motor tasks rather than added alongside them. This configuration has been identified as particularly effective for enhancing adaptive behavior in informationally rich environments [17]. This pursuit of a high information rate must be reconciled with the fall risk inherent to physically engaged movement in immersive three-dimensional environments, a concern of particular relevance for healthy older adults. MAGIC design addresses this concern at the hardware and interaction level rather than by reducing informational richness: motor-cognitive bricks involving locomotion or high force production are preferentially delivered in seated or semireclined configurations (as in the recumbent-bike Silver Explorer prototype discussed below), and mixed reality passthrough, rather than fully occluded VR, is favored for daily domestic use because it preserves the user’s perceptual awareness of the surrounding physical space throughout the experience. The cascade of formats discussed later in this paper, from supervised location-based installations to lightweight passthrough mixed reality for daily use, is itself a safety architecture as much as an accessibility one.

## Semantic, Narrative, and Motor-Cognitive Engagement

In MAGIC exergames, participants navigating a virtual heritage site are not performing a motor task and a cognitive task in parallel; instead, they are inhabiting a world whose exploration is simultaneously sensorimotor, semantic, episodic, and aesthetic. These multimodal, semantically dense, and motor-cognitively demanding environments are lacking in most available exergames, which are focused on gamified exercise libraries rather than meaningful cultural worlds. Several studies have demonstrated that most brain structures vulnerable to aging are those recruited when older adults navigate a culturally rich, narratively coherent environment, which continuously engages episodic memory (recognizing historical objects), semantic memory (retrieving contextual knowledge), spatial navigation (orienting within an immersive scene), and prospective cognition (anticipating what comes next in the narrative) [36,37]. Recently, Coronel-Oliveros et al [22] published the first large-scale demonstration of the link between creative engagement and measurable protection of brain health through delaying functional aging of the involved cerebral networks. It should be noted that Coronel-Oliveros et al [22] documented a dose-dependent effect of active creative practice and expertise (dance, music, visual arts) rather than receptive exposure to a predesigned cultural environment. However, neurobiological evidence for receptive cultural engagement specifically comes from the epidemiological literature. For instance, Tymoszuk et al [38] showed that sustained, repeated engagement with theater, concerts, and museums was associated with improvements in well-being. Moreover, Fancourt et al [39], using epigenetic clocks in 3556 adults from a nationally representative UK cohort, provided the first biological evidence that arts and cultural engagement, including both participatory and receptive activities (attending performances, visiting heritage sites, reading, making music), is associated with a 2% to 4% slower pace of biological aging, in a dose-dependent fashion comparable in magnitude to physical activity. These studies directly support the hypothesis that receptive cultural immersion, of the kind delivered by MAGIC exergames, engages neurobiological mechanisms productive for healthy aging, independently of active creative practice. The ACM provides the complementary neurobiological rationale to the potential benefits of narrative, culturally, and esthetically rich environments for brain and cognition. It proposes that the simultaneous engagement of complex motor skills, physical activity, and higher-order cognitive functions in adaptive, goal-directed motor behavior activates neuroplastic mechanisms more effectively than either domain alone. Based on this model, motor-cognitive exergaming, in which complex motor sequences are simultaneously combined with incorporated cognitive demands (moving while thinking), is hypothesized to produce superior cognitive benefits to either physical-cognitive dual-task arrangements or single-domain training, even when the intensity of physical activity is low [9,17,35].

## Emotional and Autobiographical Resonance

Cultural content anchored in the biographical and historical world of current older adults carries a neurobiological asset that purposive exergames entirely lack, that is, autobiographical resonance. Music from one’s youth, heritage sites from one’s region, artworks of personal significance—these stimuli engage the emotional-valuation system with an intensity that generic game content cannot match. According to the triad model proposed by Chatterjee and Vartanian [19], aesthetic experience simultaneously activates sensorimotor, emotion-valuation, and meaning-knowledge neural systems. Moreover, the physiological and affective “chills” triggered by music or visual art are presumably associated with dopaminergic [36], creating a pleasure signal that the TEMPA framework identifies as the most powerful predictor of motivation and sustained participation [37]. This emotional engagement consolidates learning, potentiates synaptic plasticity, and biases future behavior toward re-engagement. A MAGIC exergame that generates genuine aesthetic emotion is assumed to create, with each session, an automatic motivational trace that draws older adults to a self-reinforcing dynamic that gamified training protocols have consistently failed to produce.

## Pleasure, Effort Minimization, and Motivational Architecture

Designing for sustained engagement in MAGIC exergames therefore requires a motivational framework that operates simultaneously at 2 levels: the reflective level of conscious goal pursuit and the automatic, prereflective level that determines whether physical activity is initiated at all. The integration of these 2 frameworks yields the motivational architecture that MAGIC exergames require, in consistency with the classification of exergaming motivational dynamics proposed by Manser et al [24]. Self-Determination Theory [40], applied to MAGIC exergames, generates useful design principles—player choice to support autonomy, adaptive difficulty to support competence, cooperative or social modes to support relatedness—which are associated with higher deliberative engagement and internalization of motivation [24]. The relatedness dimension deserves further development than the present model currently affords, since the MAGIC framework as developed above focuses primarily on the individual’s interaction with the cultural environment, while social engagement is independently established as protective against cognitive decline. Plausible avenues for incorporating a social dimension without displacing the individually paced, narratively open structure of Cultural Snacking include asynchronous shared exploration of the same cultural world, in which players leave discoverable traces or annotations for others who visit later, and noncompetitive collaborative narrative milestones reached jointly across a small group’s cumulative individual sessions. These remain design proposals rather than validated mechanisms and are noted here as a direction for future elaboration of the model. However, this framework, which operates exclusively within the reflective motivational system, cannot account for the automatic barrier that precedes deliberate engagement. This limitation is addressed by the TEMPA [41,42], which demonstrates that movement-related cues are automatically processed as costly, that is, as an aversive signal that the brain evaluates and acts upon before any deliberate motivational reasoning is engaged. Exergames whose training logic is perceptually transparent are particularly vulnerable to TEMPA dynamics. Cultural immersion offers a fundamentally different architecture in this respect: a senior navigating a virtual heritage site is not experiencing effort; they are living an adventure. In the vocabulary of Csikszentmihalyi’s flow theory [43], the challenge-skill balance is naturally calibrated by the cultural content itself, and the loss of self-consciousness that characterizes flow is precisely the condition under which automatic effort minimization is most effectively suspended. Converging evidence confirms that pleasure experienced during physical activity, not in anticipation, not afterward, is the strongest predictor of long-term adherence [41] and that individualized prescriptions targeting moment-to-moment affective valence generate higher adherence rates than standard prescriptions at 8-week follow-up [42]. In the language of TEMPA, cultural immersion does not merely motivate; it restructures the automatic cost-benefit calculation that determines whether the activity happens at all.

Thus, MAGIC exergames are distinct from conventional approaches to exergames in which motivation is engineered through gamification, considered as an extrinsic incentive architecture superimposed upon a training activity to make it more tolerable. In MAGIC exergames, gamification is not the solution to the motivational problem. Instead, the cultural experience, the narrative world, the aesthetic resonance, and the autobiographical significance of the content generate intrinsic motivation that requires no external scaffolding, because inhabiting a world that matters is already what the player desires.

The heterogeneity of motivational profiles among healthy older adults requires that this integrated framework be operationalized across three distinct engagement pathways, each corresponding to a different relationship between the player and exergaming, and each instantiating a different mode of engagement [22] (see Table 1). The first pathway concerns players motivated primarily by the intrinsic pleasure of cultural exploration and narrative discovery, rather than by any explicit improvement goal. Unlike in conventional exergames, where gamification mechanics (points, rewards, levels) provide the motivational scaffold, in MAGIC exergames the cultural experience constitutes the primary motivational substrate. Accordingly, engagement corresponds to *acti-gaming* [24], that is, occasional, curiosity-driven interaction in which physical and cognitive activity is a natural consequence of cultural immersion rather than a deliberate training investment. The design priority for this pathway is brief, narratively open episodes that generate voluntary return through incompleteness and aesthetic resonance rather than through reward accumulation. Attention is absorbed by the cultural world, and the physical activity embedded within it is experienced not as effort but as the natural condition of inhabiting an experience the player actively desires. The second pathway concerns players who return regularly not to improve but to sustain their capacities through the pleasure of cultural immersion, which could be termed “cultural discovering while exercising.” Here the distinction from classical exergames is sharp: the player is not returning because a gamification system incentivizes them to do so, but because an unresolved narrative, an esthetically compelling environment, or an autobiographically resonant cultural world draws them back intrinsically. Their engagement corresponds to *exergaming proper*. Because these players are not motivated by explicit progress, failure must be rare and brief, and progression must remain invisible as training load, experienced instead as deepening cultural and aesthetic richness. The design priority is narrative coherence and continuity across sessions. The cultural world must persist, evolve subtly between episodes, and generate the sense that something awaits one’s return. The third pathway concerns players who are explicitly motivated by improvement of functional capacities and who seek in the MAGIC exergame an opportunity in this respect. Even for these players, the MAGIC framework proposes that the challenge structure be embedded within the cultural narrative rather than superimposed upon it as an explicit training layer. Challenges are presented as culturally situated opportunities that require precise motor sequences, demand spatial navigation under cognitive load, or call for sustained attentional engagement, so that the training logic remains legible to those who seek it while remaining transparent to those who do not. Their engagement corresponds to *exergame-based training* [22]. For these players, gamification mechanics may play a supporting role, but the primary motivational substrate remains the cultural experience and the narrative world—with gamification serving as a complementary layer rather than the foundational architecture.

## Rethinking the Use of Exergames: Density, Intensity, and Cultural Snacking

To ensure their effectiveness in preserving brain health and cognition, a critical design challenge for MAGIC exergames concerns the translation of principles of exercise sciences into a format compatible with the lived reality of healthy older adults. Sustained neuroplastic benefit requires repetition, progressive challenge, and sufficient accumulated volume. Immersive cultural experiences are usually delivered as location-based events, typically designed as singular, unrepeatable events. Therefore, the question is how to make exergame formats more culturally appealing and how to reconceive the architecture of engagement itself. Two converging bodies of evidence may help to reframe this. First, Herold et al [44] demonstrated that the standard Frequency, Intensity, Time, Type framework is insufficient to characterize the dose-response relationship between physical activity and brain health, and that *density*—the temporal distribution of activity bouts across the day—must be treated as an independent and potentially decisive variable. Second, the exercise snacking literature shows that brief, intermittent bouts of physical and cognitive activity distributed across the day produce cognitive benefits at least comparable to, and in some respects superior to, equivalent continuous volumes, with an optimal accumulated daily duration of approximately 90 minutes best achieved through multiple short bouts rather than extended single sessions. These findings are directly transformative for MAGIC design. Rather than architecting sessions around conventional exercise prescriptions, MAGIC exergames should be conceived as high-density, narratively serialized micro-episodes whose temporal distribution and cultural continuity across the day become active design variables.

Regarding intensity, the conventional assumption that brain plasticity and cognitive benefits require moderate-to-vigorous aerobic effort is substantially qualified when movement occurs in informationally rich environments with complex motor-cognitive demands. Carrubba et al [17] demonstrated that even moderate- or low-intensity physical activity produces neuroplastic effects comparable to high-intensity training when embedded in rich environments, because it is the continuous adaptive negotiation between a moving body and a demanding environment, not cardiovascular intensity per se, that drives the neuroplastic response. What MAGIC exergames require is not a high heart rate but a high information rate. This opens MAGIC to the full spectrum of mobility and fitness levels characteristic of healthy older adults, without requiring the aerobic effort that purposive exergames demand and that TEMPA dynamics make so difficult to sustain. The design principle that synthesizes both insights is what we term *Cultural Snacking*, which consists of the serialization of MAGIC content into autonomous episodes of 5 to 10 minutes, each complete in itself yet narratively open, designed for 3 to 5 daily consumptions across varied contexts—home, residential facility, hotel, workplace—with or without full VR immersion. The mechanism of re-engagement in Cultural Snacking is categorically different from the gamification logic that underlies conventional exergame design. In gamified exergames, re-engagement is engineered through extrinsic incentive structures—point accumulation, streak rewards, leaderboard positioning, unlockable content—that create artificial obligations to return. Cultural Snacking operates on an entirely different motivational logic. Re-engagement is driven by narrative incompleteness and aesthetic absorption to continue a story that matters to players—a heritage site not yet fully explored, a historical figure whose motives remain unresolved, a cultural object whose significance has only been partially revealed. This is intrinsic motivation in its purest form; it requires no external scaffolding because the activity itself is the reward. It is precisely the motivation that cultural content is uniquely positioned to generate in an older adult population for whom personal history, aesthetic experience, and cultural resonance carry a depth of meaning that points, badges, and leaderboards cannot replicate. Critically, progression in Cultural Snacking does not occur through the explicit escalation of cognitive task difficulty. Instead, progression operates entirely through the gradual modification of environmental constraints, in the spirit of ecological dynamics: richer spatial configurations, more ambiguous cultural affordances, temporally more demanding motor-cognitive sequences, as the narrative world deepens across episodes. This progression is invisible as training load but experienced as increasing narrative and aesthetic richness. The player does not feel themselves becoming more capable—they feel the world becoming more meaningful. The neuroplastic benefit and the cultural experience are not merely adjacent: they are constitutive of each other. Between episodes, the cultural world persists and subtly evolves, generating the sense that something awaits one’s return. In the framework of TEMPA, this mechanism is hypothesized to convert the automatic cost evaluation of physical effort into an automatic invitation. A legitimate concern is whether the repeated transition between daily activities and an immersive device, several times a day, would itself impose a cognitive and logistical load that conflicts with the effort-minimization principle the model otherwise seeks to exploit. We argue that this friction is directly addressed by the choice of delivery hardware rather than by the Cultural Snacking architecture itself: next-generation standalone mixed reality headsets require no external computer or cabling, start through a single gesture, and, in passthrough mode, do not require play-space recalibration between sessions. This remains a design hypothesis rather than an established finding, and we return to it in the research agenda below, where repeated-engagement friction is identified as a measurable outcome in its own right [44]. The exercise snacking evidence [45] and the Cultural Snacking model establish that MAGIC exergames are not merely more engaging than conventional exergames—they are more neuroplastically effective and more motivationally coherent.

## Conclusion and Perspectives

Based on underwhelming research on the hypothesized superiority over conventional alternatives. This Viewpoint paper recommends adopting a fundamentally different conceptual approach to designing MAGIC exergames: (1) targeting cognitively healthy older adults as the primary prevention population, (2) grounding their design in the neurobiological logic of HMI and Ecological Dynamics, and (3) placing culturally meaningful, aesthetically rich, and autobiographically resonant content at the center of the intervention. Moreover, the mechanistic pillars of MAGIC exergames developed in the present paper converge on a single design principle that inverts the classic logic of exergame design by assuming that in MAGIC exergames, pleasure is not a reward added to a training protocol: it is the training protocol. A practical challenge that this approach must confront is scalability: content that is culturally rich and personally meaningful risks becoming prohibitively costly to produce across different regions, languages, and population subgroups if each MAGIC environment is built as a bespoke production. Two factors are likely to mitigate this constraint. First, the motor-cognitive bricks specified by the combined-training taxonomy are largely reusable across narrative settings, so that production cost concentrates in environmental and narrative content rather than in the validated interaction mechanics themselves. Second, partnerships with heritage and cultural institutions, which already hold digitized or digitizable collections, offer a route to amortizing content production beyond what a single research or commercial actor could sustain alone. Whether this is sufficient to keep MAGIC content affordable at population scale remains an open empirical and economic question.

Practically, MAGIC exergames could be conceived as a system of complementary formats—from location-based event to the domestic sphere, organized along the reality-virtuality continuum, each optimal for a different use context, immersion depth, and accessibility level, yet all anchored in the same narrative and cultural world. Large-scale projection environments without headsets offer high aesthetic immersion with zero technological barrier, making them ideal for weekly location-based MAGIC sessions. Augmented reality on tablets and smartphones provides a zero-barrier entry point for the shortest micro-snacks integrated into daily life. Mixed reality passthrough devices represent the most promising format for daily domestic Cultural Snacking: immersive enough to sustain narrative engagement, safe enough to eliminate fall risk, and sufficiently lightweight for repeated daily use. This cascade architecture resolves simultaneously the cybersickness problem, the repetition problem by providing lightweight formats compatible with Cultural Snacking, and the accessibility problem by allowing older adults of very different technological comfort levels to enter the same cultural world through different doors. An advanced existing proof-of-concept for this convergence is *Submersive* (Austin, Texas; projected opening 2027), which is explicitly positioned at the intersection of immersive art, neuroaesthetics, and therapeutic wellness, while integrating wearable biometric sensors to measure in real time how immersive artistic environments affect the human body and brain, feeding data back into iterative space design. *Submersive* thus illustrates, as a project still under development rather than as established evidence, the convergence this paper advocates: a private operator from the entertainment world deliberately integrating the conceptual tools of neuroaesthetics and health research to transform a cultural experience into a wellness intervention. Should it open as planned and meet its stated design objectives, Submersive would provide further commercial validation of this convergence; at present, however, it remains a projected outcome rather than a demonstrated one, a distinction this paper is careful to maintain given its own critique, in earlier sections, of unrealistic optimism in the exergaming field.

What it still lacks to constitute the full MAGIC model is an explicit research agenda. This could be structured around three main recommendations. First, future studies must abandon the “technology-first approach” to characterize MAGIC exergames by the type of combined motor-cognitive interactions they deliver—specifying the physical intensity, motor challenge, cognitive load, and cultural content richness as independent and measurable variables. This requires the development of a dedicated taxonomy that incorporates dimensions of narrative coherence, aesthetic richness, and autobiographical resonance as primary design variables. Second, mechanistic investigations are needed, including neuroimaging to identify longitudinally sensitive biomarkers of preventive efficacy and neuroplastic change that creative cultural engagement is expected to produce. Third, comparative designs with active control groups—conventional exergames, standard combined training, and real-world cultural engagement—with adequate follow-up periods are necessary to establish both the superiority and the persistence of MAGIC-induced benefits. Concrete, measurable outcomes that would distinguish a MAGIC intervention from a standard multidomain exergame in a controlled trial should include functional brain-age indices derived from network-level connectivity measures, of the kind reported by Coronel-Oliveros et al [22]. For instance, electroencephalographic comparison between a MAGIC condition and a content-matched “library” condition presenting the identical cognitive-motor bricks without narrative or cultural framing could isolate the neurophysiological contribution of cultural embedding; spontaneous re-engagement rate could serve as a behavioral proxy for intrinsic adherence, which directly distinguishes the MAGIC motivational model from approaches reliant on extrinsic gamification incentives. These outcomes should also be tracked against the logistical-friction hypothesis raised above regarding repeated daily device use.

The future development of MAGIC exergames requires an intersectoral and multidisciplinary innovation ecosystem. The raw material of MAGIC resides in cultural institutions, while the neurobiological and design knowledge required to translate this content into effective interventions resides in digital health research teams. Bringing these 2 communities into structured co-design programs, with older adults as co-designers, is the essential first institutional step. Indeed, location-based immersive cultural experiences, which already attract a predominantly senior audience, represent a natural entry point for proof-of-concept studies, combining high-quality cultural content with physical engagement in ecologically valid, nonclinical settings. Thus, without waiting for MAGIC-specific trials, we invite the digital medicine, public health, and silver economy communities to recognize that cultural and artistic content is potentially the most ecologically valid, intrinsically motivating, and cost-effective setting for a new generation of immersive exergaming interventions that are as meaningful as they are effective in enhancing brain health, cognition, and well-being. The scientific, institutional, and economic conditions for this convergence already exist.

## Abbreviations

ACM: Adaptive Capacity Model
HMI: Human Movement Intelligence
MAGIC: Motor-Cognitive Active Gamified Immersive Cultural
TEMPA: Theory of Effort Minimization in Physical Activity
